# DEVELOPING DEMENTIA-SPECIFIC PROVIDER TRAINING IN MENTAL HEALTH: WHAT IS THE TARGET?

**DOI:** 10.1093/geroni/igz038.1390

**Published:** 2019-11-08

**Authors:** Mary F Wyman, Corrine I Voils, Ranak B Trivedi, Carey E Gleason

**Affiliations:** 1 WS MIDDLETON MEMORIAL VETERANS HOSPITAL, Madison, Wisconsin, United States; 2 WS Middleton Memorial Veterans Hospital, Madison, Wisconsin, United States; 3 Stanford University/Palo Alto VA, Menlo Park, California, United States; 4 W.S. Middleton Memorial Veterans Hospital, Madison, Wisconsin, United States

## Abstract

Most persons with dementia (PwD) live in the community and receive mental health care in the outpatient setting, making these providers an important target for education to improve dementia care. To inform the development of training curricula, we surveyed 65 mental health providers in a Veterans Affairs outpatient clinic on perceived barriers and training needs related to service delivery to PwD and caregivers. We used an adapted version of the Sense of Competence in Dementia Care Staff scale to assess domain-specific competencies. Respondents rated this work as highly important and wanted dementia-related training. They reported low competency in person-centered care approaches and challenging clinical situations, e.g., managing risk of harm. System-level barriers affecting services for PwD were noted. Findings suggest that outpatient mental health professionals are an underutilized resource in dementia care. This work can inform the development of provider training and identification of system-level barriers in this setting.

